# Biodegradation of butyronitrile and demonstration of its mineralization by *Rhodococcus* sp. MTB5

**DOI:** 10.1007/s13205-016-0456-0

**Published:** 2016-06-22

**Authors:** Ismailsab Mukram, Masarbo Ramesh, T. R. Monisha, Anand S. Nayak, T. B. Karegoudar

**Affiliations:** Department of Biochemistry, Gulbarga University, Kalaburagi, 585106 Karnataka India

**Keywords:** Butyronitrile, *Rhodococcus* sp. MTB5, Complete degradation, Metabolite feeding

## Abstract

A nitrile utilizing bacterium *Rhodococcus* sp. MTB5 was previously isolated in our laboratory by the enrichment culture technique. It is able to utilize butyronitrile as sole carbon, nitrogen, and energy source. Maximum butyronitrile degrading property of this strain has been investigated. Results reveal that 100, 98, and 88 % degradation was achieved for 2, 2.5, and 3 % butyronitrile, respectively. The strain is capable of growing in as high as 5 % butyronitrile concentration. A two-step pathway involving nitrile hydratase (NHase) and amidase was observed for the biodegradation of butyronitrile. Complete degradation (mineralization) of butyronitrile with the help of metabolite feeding experiment was reported. The significance of this investigation was the capability of the strain to completely degrade and its ability to grow on higher concentrations of butyronitrile. These potential features make it a suitable candidate for practical field application for effective in situ bioremediation of butyronitrile contaminated sites.

## Introduction

Nitriles are the cyanide containing compounds (R–CN) and are widespread in the environment as a result of biological and industrial activity (Legras et al. 1990). They are used as preliminary materials for the synthesis of a numerous fine chemicals (Banerjee et al. 2002). Butyronitrile is an aliphatic nitrile, clear colourless liquid with a suffocating odour resembling bitter almond oil. It is miscible with most polar organic solvents, hence finds applications in the industries in making other chemicals. It is mainly used as a precursor to the poultry drug amprolium (Peter et al. 2002). Amprolium is a coccidiostat (antiprotozoal agent) used in the poultry that acts upon coccidia parasites. Amprolium is prepared using commercially available butyronitrile as starting material. It is also used in electrolyte composition in dye-sensitized solar cells (Sauvage et al. 2011). A number of nitriles have been reported as potent carcinogenic, mutagenic, and toxic in nature (Ramakrishna et al. 1999). The use of nitriles as bulk solvents has increased their distribution in the environment and needs their remediation (Ebbs 2004). The chemical hydrolysis of these compounds necessitates harsh conditions, such as extremes of pH and elevated temperatures with the creation of significant quantities of by-products and secondary pollutants (Kobayashi and Shimizu 2000; Prasad et al. 2009). Bioremediation is one such method used for the detoxification of contaminated sites. It is an inexpensive technology, and can either eliminate these compounds by degrading them to harmless intermediates or, in due course, to carbon dioxide and water (Nawaz et al. 1991).

**Fig. 1:**
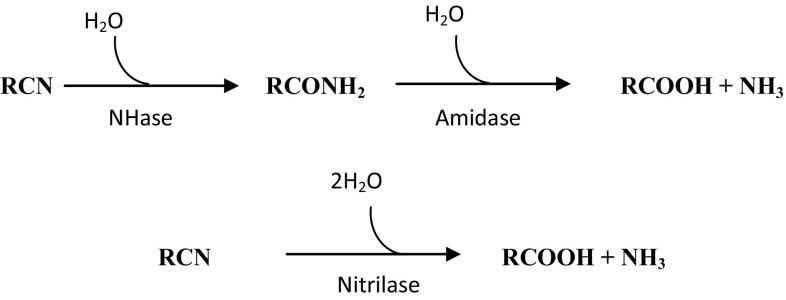
General catabolic pathways (NHase: amidase and nitrilase) for degradation of organic nitriles

The nitrile converting enzymes occur in a wide variety of plants, bacteria, and in some fungi. The microbial hydrolysis of nitriles begins through two key enzymatic pathways (Fig. 1). First, the nitrile hydratases hydrolyze nitriles to amides and by amidases to the corresponding carboxylic acids and ammonia (Kobayashi and Shimizu 1998; Sharma et al. 2013). Second, nitrilases directly convert nitriles to acids and ammonia (Kobayashi and Shimizu 2000).

Microbial degradation of different types of nitriles has been reported using different strains (Wang et al. 2007; Chen et al. 2010; Fang et al. 2015). However, there are limited reports in the literature about the biodegradation of butyronitrile (Wenzhong et al. 1991; Nawaz et al. 1992). To the best of our knowledge, no reports are available on the proof of complete degradation of nitriles by metabolite feeding experiment. In the present communication, we report the maximum degradation of butyronitrile of 3 % and the ability of the strain to grow in a mineral salts (MS) medium containing as high as 5 % butyronitrile. This is the first report on the highest degradation of any nitrile reported and the confirmation of complete degradation by metabolite feeding experiment.

## Materials and methods

### Bacterium, media, and growth conditions

*Rhodococcus* sp. MTB5 used in the present study was previously isolated in our laboratory (Mukram et al. 2015). For butyronitrile degradation studies, the bacterium was grown in MS medium supplemented with butyronitrile as carbon and nitrogen source. Medium composition was the same as explained previously (Mukram et al. 2015). The pH of the medium was adjusted to 7.0 using 2 N NaOH or 2 N HCl. Cells were grown in 250 ml Erlenmeyer flasks with shaking at 180 rpm in orbital shaker at 30 °C.

### Butyronitrile degradation

The degradation of butyronitrile was carried out for freely suspended cells of *Rhodococcus* sp. MTB5. A series of batch experiments were carried out in 250 ml flasks containing 50 ml of autoclaved medium loaded with different concentrations of filter sterilized butyronitrile (2, 2.5, and 3.0 % v/v) and with suitable controls. The butyronitrile grown *Rhodococcus* sp. MTB5 free cells in exponential growth phase (6.8 × 10^9^ CFU/ml) were used as inoculum. The degradation process was carried out on a rotary shaker at 30 °C for 12, 24, 36, 48, 72, 84, and 96 h of incubation periods. All experiments were conducted in triplicate. Samples from the culture broth were withdrawn under sterile conditions at the above indicated time intervals to check the growth of the organism and for the analysis of residual butyronitrile. Butyronitrile % degradation, $\eta$, at different time periods of incubation was defined by the following expression:

$${\text{Degradationratio}},{\mkern 1mu} \eta {\mkern 1mu} {\mkern 1mu} (\% ) = \frac{{S_{0} - S}}{{S_{0} }} \times 100$$

where *S*_0_ represents the initial butyronitrile concentration (%) prior to biodegradation; *S* represents the residual butyronitrile concentration (%) after degradation.

### Estimation of ammonia release

Degradation of butyronitrile was even measured in terms of estimation of ammonia, as it is the indicator of nitrile cleavage and it is one of the end products in catabolism of nitriles. Production of ammonia from butyronitrile hydrolysis was quantitatively determined in the culture filtrate according to Vogel (1969).

### Preparation of cell-free extract

The cells were harvested at mid-log growth phase by centrifugation at 8000*g* for 10 min at 4 °C and washed twice with 100 mM phosphate buffer (pH 7.0). The cell-free extract was prepared by following the procedure of Veeranagouda et al. (2006).

### Enzyme assays

NHase activity was assayed in a reaction mixture comprised an appropriate amount of cell-free extract, 25 mM butyronitrile and 100 mM phosphate buffer pH 7.0. The reaction was administered at 37 °C for 15 min and arrested by adding 1 M HCl. The mixture was then centrifuged at 8000*g* for 10 min, and the resulting supernatant was used for the analysis of butyramide by GC. One unit of NHase activity was defined as the amount of enzyme catalysing the formation of 1 μmol of butyramide per min under assay conditions.

The amidase activity was assayed in a reaction mixture containing phosphate buffer (100 mM, pH 7.0), 50 mM butyramide, and appropriate amount of cell-free extract and incubated for 30 min at 37 °C in a water bath. The reaction was stopped by the addition of 1 M HCl and centrifuged. The amount of ammonia released was measured according to Schär et al. (1986). One unit of amidase activity was defined as the amount of enzyme catalysing the formation of 1 μmol of ammonia per min.

### Metabolite feeding experiments

The butyramide and butyric acid are the two metabolic intermediates of butyronitrile degradation (Mukram et al. 2015). For further confirmation of complete degradation of butyronitrile, we have checked the ability of the organism to utilize these intermediate metabolites as sole source of carbon and/or nitrogen. Feeding experiment was carried out by supplying these metabolites to the strain MTB5 as growth substrates in 250 ml flasks containing 50 ml MS media. The media used for the butyric acid feeding study were supplied with the 1 mg ml^−1^ of NH_4_NO_3_ as the nitrogen source. Butyronitrile grown cells (5 % v/v culture) were harvested by centrifugation at 8000*g* for 10 min and washed with phosphate buffer used as inoculum. The flasks were incubated on shaker at 30 °C, simultaneously incubated culture flasks without metabolites as well as uninoculated flasks containing metabolites served as controls. All these experiments were performed in triplicates. The growth of the bacteria, residual butyramide, and butyric acid concentration was determined.

### Growth of *Rhodococcus* sp. MTB5 on increasing butyronitrile concentrations

To five 250 ml flasks containing 50 ml of MS medium, different concentrations of butyronitrile (3.5–5.5 %) were supplemented and the flasks were inoculated with exponential growth phase cells of strain MTB5. All the flasks were incubated in shaker at 30 °C, and the growth was determined at regular intervals of time.

### Analytical methods

Bacterial growth was determined spectrophotometrically at 660 nm (analytikjena-SPECORD 50). Butyronitrile, butyramide and butyric acid concentrations were determined by gas chromatography (GC). The GC model used and the chromatographic conditions followed were as described previously (Mukram et al. 2015).

## Results

### Butyronitrile degradation

**Fig. 2:**
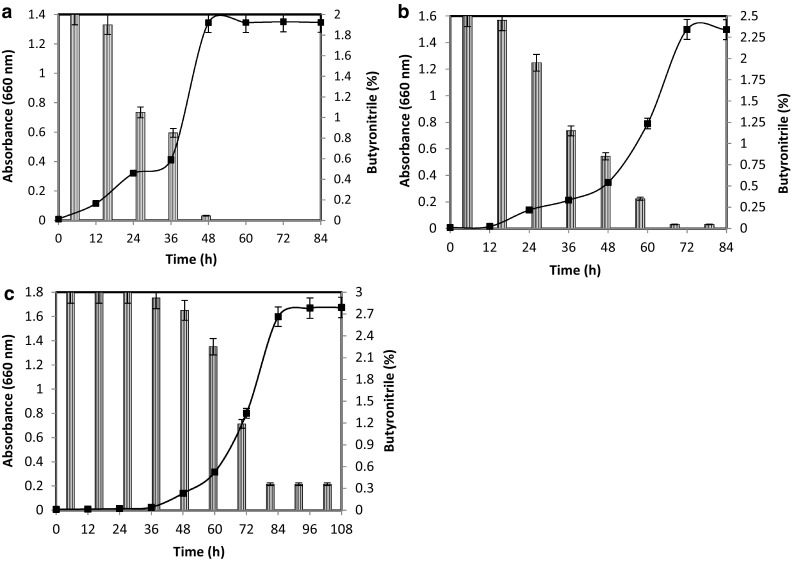
Growth of *Rhodococcus* sp. MTB5 and degradation of 2 % (**a**), 2.5 % (**b**) and 3 % (**c**) butyronitrile. Growth (*filled squares*); butyronitrile (*dashed columns*)

**Fig. 3:**
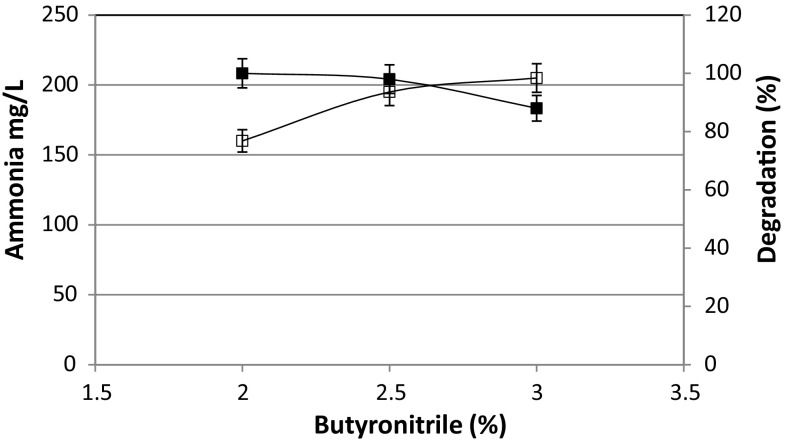
Amount of ammonia (*open squares*) released and  % degradation (*filled squares*) of 2, 2.5, and 3 % butyronitrile by *Rhodococcus* sp. MTB5

2.0 % of butyronitrile was completely degraded within 60 h of incubation, and at the same period, the strain attained its maximum growth (A_660_ nm, 1.345) (Fig. 2a). Maximum growth was observed at 72 h of incubation (A_660_ nm, 1.499) when *Rhodococcus* sp. MTB5 was grown in MS medium with 2.5 % butyronitrile as the sole carbon and nitrogen source. The strain showed 98 % utilization of 2.5 % butyronitrile (Fig. 2b) within 72 h. When the butyronitrile concentration was further increased to 3.0 %, maximum growth of the organism was seen at 96 h of incubation (A_660_ nm, 1.669) and showed 88 % utilization of 3.0 % butyronitrile (Fig. 2c). No further degradation of 3.0 % butyronitrile was observed even after prolonged incubation. The free cells have successfully degraded butyritrile within 60 h of incubation when the concentration of butyronitrile was 2.0 % (v/v). When nitrile concentration was increased to 2.5 and 3.0 %, the rate of degradation was slightly decreased. Furthermore, when its concentration was increased beyond 3 %, the degradation efficiency was further decreased (data not shown). Furthermore, the higher concentration of butyronitrile adversely influenced the viability of the cells. In the control experiment, the concentration of butyronitrile remained the same in uninoculated culture flasks containing butyronitrile. This confirmed the utilization of butyronitrile as a consequence of hydrolysis of butyronitrile by *Rhodococcus* sp. MTB5. The concentration of ammonia was estimated at various time intervals with the maximum accumulation of 160, 195, and 205 mg l^−1^ for 2, 2.5, and 3 % butyronitrile degradation, respectively (Fig. 3).

### Enzyme assays

NHase and amidase are the two enzymes involved in the butyronitrile degradation in *Rhodococcus* sp. MTB5 were assayed. The NHase activity was found to be 0.194 µmol of butyramide per min of incubation per ml of cell-free extract, whereas amidase activity was calculated to be 0.524 µmol of ammonia per min of incubation per ml of cell-free extract.

### Feeding experiments

**Fig. 4:**
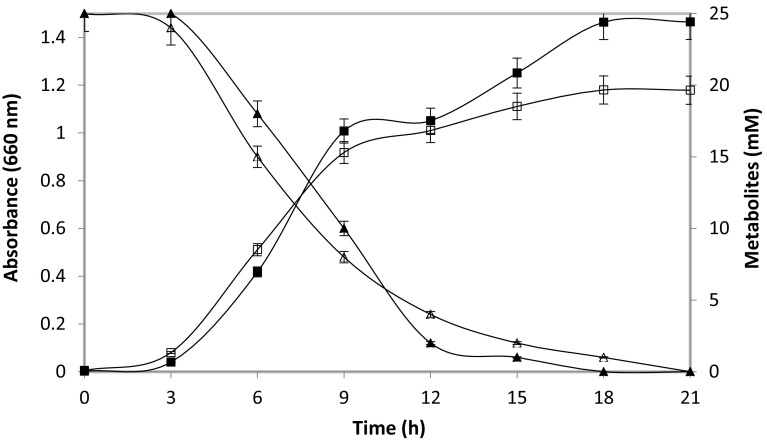
Growth of *Rhodococcus* sp. MTB5 on metabolites, butyramide, and butyric acid, and their utilization. Growth on butyramide (*open squares*) and butyric acid (*filled squares*); utilization of butyramide (*open triangles*) and butyric acid (*filled triangles*)

**Fig. 5:**
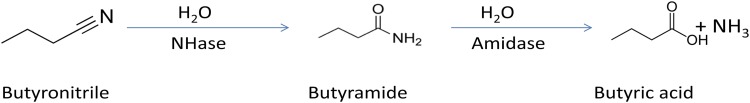
Catabolic pathway of butyronitrile in *Rhodococcus* sp. strain MTB5

Both the metabolites were found to be efficiently metabolized by the strain (Fig. 4). When the strain was grown in MS medium provided with butyramide as carbon and nitrogen source, the organism attained the maximum growth within 18 h of incubation. The maximum growth was indicated by optical density at 660 nm wavelength as 1.181, and then, it entered stationary growth phase. It completely utilized butyramide in 21 h of incubation. The growth of the strain was also investigated in the presence of butyric acid. Strain reached stationary phase in 21 h, and the maximal optical density at 660 nm wavelength was 1.465. Butyric acid was completely utilized by MTB5 in 18 h of incubation. These observations confirm the ability of *Rhodococcus* sp. strain MTB5 to utilize butyronitrile metabolites. Based on the results obtained, the butyronitrile degradation in *Rhodococcus* sp. MTB5 follows a bi-enzymatic NHase and amidase pathway (Fig. 5).

**Fig. 6:**
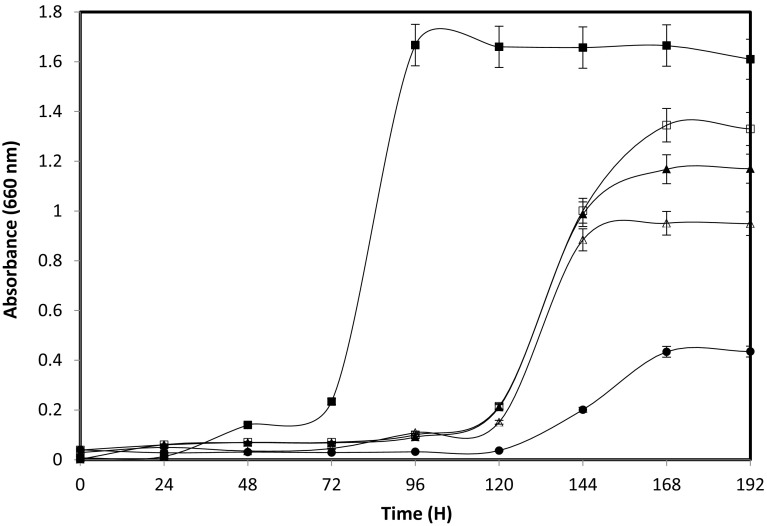
Growth of *Rhodococcus* sp. MTB5 on increasing concentrations of butyronitrile: 3.5 % (*filled squares*), 4 % (*open squares*), 4.5 % (*filled triangles*), 5 % (*open triangles*) and 5.5 % (*filled circles*)

Growth of *Rhodococcus* sp. MTB5 was investigated at increasing concentrations of butyronitrile (Fig. 6). The highest growth was observed at 3.5 % butyronitrile concentration, and above this concentration, the growth of the bacterium decreased gradually. However, concentrations above 4 % induced a prolonged lag phase in bacterial growth. Conversely, beyond 5.5 % of butyronitrile growth was completely suppressed.

## Discussion

As per the available literature, the degradation of aliphatic nitriles follows through NHase and amidase pathway (Santoshkumar et al. 2011). Large numbers of reports are available on the degradation of aliphatic nitriles and most of them are on acetonitrile (Sorokin et al. 2007; Manolov et al. 2005; Feng and Lee 2009). However, only limited reports are available on the degradation of butyronitrile (Wenzhong et al. 1991; Nawaz et al. 1992), and among them, most reports are on the growth of the microorganisms on butyronitrile as carbon and/or nitrogen source (Nawaz et al. 1989; Linardi et al. 1996; Kao et al. 2006).

Nawaz et al. (1992) reported the NHase and amidase catalysed degradation of 36 mM of butyronitrile by *Klebsiella pneumonia* in 96 h of incubation. Two strains of bacteria, *Corynebacterium boffmanii* and *Arthrobacter flavescens*, were reported for the 100 % removal of butyronitrile of concentration 10 g l^−1^ in 12 and 36 h of incubation, respectively (Wenzhong et al. 1991). *Pseudomonas putida* was reported to grow on 0.2 % butyronitrile as a sole source of carbon and nitrogen, and the utilization was shown in terms of accumulation of ammonia (Nawaz et al. 1989). Kao et al. (2006) reported the effects of butyronitrile concentration (25, 50 and 100 mM) on the growth of *Klebsiella oxytoca* where in, 100 mM butyronitrile induced an apparent lag phase in bacterial growth and its rate of degradation decreased and the time required was around 125 h. *Burkholderia cepacia* strain C-3 capable of degrading butyronitrile was demonstrated in terms of growth on butyronitrile, as carbon and nitrogen source was reported by Adjei and ohta (1999). Kaur et al. (2014) isolated nitrile-hydrolysing bacterium *Isoptericola variabilis* RGT01 and showed its hydrolysing ability against 100 mM butyronitrile. Acetonitrile and benzonitrile grown terrestrial and deep-sea actinomycete isolates were reported for having the butyronitrile hydrolyzing activities and the ability of butyronitrile as growth substrate for these isolates (Brandão and Bull 2003). The *Candida famata* isolated by Linardi et al. (1996) was able to grow on 0.7 % butyronitrile, as nitrogen source and ammonia concentration was detected in the supernatant. On the other hand, in the present communication, we have reported the 100, 98, and 88 % degradation of 2, 2.5, and 3 % butyronitrile, respectively, by *Rhodococcus* sp. MTB5. The utilization was further confirmed by the accumulation of ammonia in the culture medium and by enzyme assay studies. To the best of our knowledge, this is the highest amount of degradation achieved for any of the nitriles by any microorganism reported so far.

Since last three decades, many studies on degradation of different types of nitriles have been carried out. All the studies reports that either nitriles will be directly converted to acids and ammonia or by the intermediate formation of amide. The amide and/or acid are detected in the spent medium during the course of nitrile degradation, and later, they will disappear (Fang et al. 2015; Kao et al. 2006; Brandão and Bull 2003; Li et al. 2007). However, no reports are available in the literature which confirms the complete degradation of given nitrile by both metabolite (amide and acid) feeding experiment. Fang et al. (2015) reported enzymatic degradation of aliphatic nitriles by *Rhodococcus rhodochrous* BX2. Furthermore, the authors showed the complete degradation in terms of utilization of carboxylic acid by this microorganism but not the amide. Similarly, the carboxylic acid degradation has been demonstrated in *Cryptococcus* sp. UFMG-Y28 (Rezende et al. 2000). In our previous studies, we reported butyramide and butyric acid as the metabolites of butyronitrile degradation (Mukram et al. 2015). In the present investigation, we have carried out metabolite feeding experiment by allowing the organism to grow on the metabolites and then estimating the residual metabolite concentration in the culture medium. The complete disappearance of amide and acid may imply that the given nitrile can be thoroughly decomposed to CO_2_ and H_2_O. Furthermore, the utilization of intermediate products, i.e., the metabolites, is a positive attribute for the bioremediation of polluted environments, because the inhibition of microbial enzyme activities by intermediate products will be reduced. Furthermore, this action will avoid the secondary pollution from by-products that usually result in negative effects (Feng and Lee 2009). Degradation of such a high concentration of butyronitrile and conformation by metabolite feeding experiment is a notable property of *Rhodococcus* sp. MTB5 and serves to be potential candidate for the removal of high amounts of butyronitrile from the industrial effluent.
